# Biologically synthesized nanoparticles: barley-mediated silver and gold nanoparticles and caged gold nanoplatform for advanced drug delivery system engineering in medicine

**DOI:** 10.1186/s11671-024-04097-3

**Published:** 2024-10-07

**Authors:** Muhammad Talaat

**Affiliations:** 1 Faculty of Pharmacy, Ain Shams University, Cairo, Egypt; 2 El Demerdash Hospital, Ain Shams University, Cairo, Egypt; 3 R&D Department, BRAND For Pharmaceutical Industries, Giza, Egypt

## Abstract

The integration of green synthesis methods and advanced nanostructure designs holds significant promise for the development of innovative nanomaterials with diverse biomedical applications. This commentary delves into the use of barley grains for the eco-friendly synthesis of silver and gold nanoparticles, highlighting their potential as biocompatible agents with potent antibacterial properties. The barley-mediated synthesis approach not only offers a sustainable and cost-effective method for producing these nanoparticles but also underscores their remarkable efficacy against pathogenic bacteria. The barley-mediated approach not only offers a sustainable and cost-effective method for producing biocompatible nanoparticles but also demonstrates remarkable antibacterial efficacy against pathogenic bacteria. By critically evaluating the strengths and potential gaps in this synthesis approach, this commentary emphasizes the importance of integrating green synthesis techniques with advanced nanoparticle applications. Future research directions should aim at optimizing synthesis processes, ensuring enhanced stability and biocompatibility, and exploring the full potential of biologically synthesized nanoparticles in medical treatments and environmental sustainability. This focus on sustainable synthesis and application could pave the way for the next generation of nanomaterials, offering significant advancements in both healthcare and ecological preservation. By examining the strengths, gaps, and potential synergies between these two approaches, this commentary underscores the importance of sustainable synthesis techniques and the development of multifunctional nanoparticles. This integrated approach could lead to the creation of next-generation nanomaterials, offering significant advancements in medical treatments and environmental sustainability.

## Introduction

The convergence of nanotechnology and green chemistry represents a pivotal development in modern scientific research, particularly within the realm of drug delivery systems. This commentary seeks to explore the intersection and implications of two pioneering studies: the green synthesis of nanoparticles using barley grains as discussed by Singh et al. [1], and the innovative development of caged gold nanostars (C-GNS) for the photothermal therapeutic applications highlighted b Canning et al. [2]. Through a comparative analysis, we aim to elucidate how these advancements not only address existing gaps in the literature but also chart a path for future research in nanoparticle synthesis and application. Green synthesis methodologies, which utilize biological resources for nanoparticle production, have garnered significant attention due to their environmentally friendly approach and potential for producing biocompatible and stable nanoparticles [1, 3]. The study by Singh et al., exemplifies this trend by employing barley grains to synthesize gold (AuNPs) and silver nanoparticles (AgNPs). This method underscores the efficiency and sustainability of using agricultural resources for nanoparticle production, highlighting the rapid synthesis and notable stability imparted by the biological corona surrounding the nanoparticles. Such innovations are crucial as they offer a viable alternative to traditional chemical synthesis methods, which are often plagued by high costs and environmental concerns. Conversely, the research by Canning et al., introduces a novel approach to nanoparticle synthesis and application, particularly in the field of theranostics and photothermal therapy. The development of C-GNS combines the exceptional plasmonic properties of nanostars with the encapsulating capabilities of hollow-shell structures, creating a versatile platform for both diagnostic and therapeutic applications. This study not only advances the field of plasmonic nanostructures but also demonstrates the potential of C-GNS in real-world biomedical applications, such as hyperspectral imaging and photothermal therapy of tumors in vivo.

The significance of these studies lies not only in their individual contributions but also in the potential synergies and gaps they reveal when considered together. Both studies address the critical need for stable, biocompatible nanoparticles with specific functional properties. The green synthesis approach of highlights the role of biological coronas in stabilizing nanoparticles and enhancing their antibacterial activity [1]. This biological interface is a key factor that could be exploited further in designing nanoparticles with targeted functionalities for drug delivery systems. On the other hand, the work on C-GNS by illustrates how structural innovations can enhance the optical and therapeutic properties of nanoparticles, providing a blueprint for future research aimed at integrating green synthesis methods with advanced nanoparticle designs. One of the primary literature gaps identified in these studies is the need for a comprehensive understanding of the interplay between the biological corona of green-synthesized nanoparticles and their functional performance in biomedical applications. While Singh et al., group provides valuable insights into the role of biological components in nanoparticle stability and antibacterial efficacy, it does not extensively explore the potential of these nanoparticles in complex biological environments or their compatibility with advanced diagnostic and therapeutic techniques like what is plotted extensively by Parveen et al., and Muntimadugu et al., [4, 5]. Conversely, Canning et al., demonstrate the sophisticated application of nanoparticles in theranostics but does not fully address the sustainability and biocompatibility of their photothermal therapeutic aspects as thoroughly as the green synthesis approach. Future research should focus on bridging this gap by investigating how the principles of green synthesis can be integrated with the advanced functional designs seen in C-GNS. This would involve exploring the potential of biologically derived stabilizing agents in enhancing the performance of photothermal nanoparticles and examining how these agents interact with different biological systems.

In contrast, the studies by Singh et al., and Canning et al., provide a solid foundation for the development of innovative and sustainable nanoparticle synthesis methods with significant biomedical applications. By critically examining the overlaps and gaps in these studies regarding the therapeutic effects and their biological properties, this commentary underscores the importance of integrating green synthesis techniques with advanced functional designs to address current challenges in nanoparticle research, in both aspects, the smart designing and diversity of the way of applications using advanced drug delivery system engineering techniques. Future work should focus on exploring these synergies to develop multifunctional nanoparticles that meet the dual goals of sustainability and high performance in medical applications.

## Main body

In the field of nanotechnology, the utilization of nanoparticles synthesized through green methods has garnered significant attention due to their potential applications in various fields, including biomedicine, environmental science, and material science. The research by Singh et al. [1] focuses on the green synthesis of gold and silver nanoparticles using barley grains, highlighting their antibacterial potential. This study demonstrates the feasibility of employing plant extracts for nanoparticle synthesis, a method that is not only eco-friendly but also cost-effective. The study employs Fourier-transform infrared spectroscopy (FT-IR), matrix-assisted laser desorption/ionization time-of-flight (MALDI-TOF) mass spectrometry, and single-particle inductively coupled plasma mass spectrometry (sp-ICPMS) to characterize the nanoparticles. The findings reveal that the barley extract contains multiple active surface groups, including proteins and amino acids, which facilitate the reduction and stabilization of gold and silver ions. The antimicrobial potential of these nanoparticles was assessed against Gram-negative bacterial strains such as Pseudomonas aeruginosa and Escherichia coli. The results show a profound antibacterial effect, with silver nanoparticles (AgNPs) exhibiting complete eradication of bacterial cells at a concentration of 8 µg/mL. This study highlights the promising application of barley-synthesized AgNPs in combating bacterial infections, a significant concern in the era of increasing antibiotic resistance.

In a parallel line of research, the development of caged gold nanostars (GNS) presents a novel approach in the realm of photothermal nanoplatforms, combining therapeutic and diagnostic capabilities within a single platform. The unique structure of these nanostars allows for enhanced plasmonic properties, making them suitable for applications in imaging, drug delivery, and photothermal therapy. One of the predominant themes in current nanoparticle research is the shift towards green synthesis methods. The use of plant extracts, such as barley grains, for nanoparticle synthesis aligns with the principles of green chemistry, emphasizing sustainability and environmental friendliness. This approach minimizes the use of hazardous chemicals and reduces the overall environmental impact of nanoparticle production. The antibacterial properties of nanoparticles, particularly AgNPs, have been extensively studied. The research indicates that nanoparticles synthesized using green methods exhibit significant antibacterial activity. This has important implications for the development of new antibacterial agents that can address the growing problem of antibiotic resistance. The ability to synthesize nanoparticles that are stable and effective against a broad spectrum of bacteria is a major advancement in this field [2].

Regarding the designing of nanoplatforms of GNS, the photothermal therapy (PTT) has emerged as a promising strategy for combating bacterial infections, particularly in the context of antibiotic resistance. PTT represents a compelling advancement in the realm of bacterial infection management, particularly in the context of the escalating global challenge posed by antibiotic-resistant pathogens. This therapeutic approach hinges on the ability of certain nanomaterials, such as AuNPs, carbon-based materials like graphene oxide, and metal–organic frameworks (MOFs), to absorb near-infrared (NIR) light and convert it into heat. Upon exposure to NIR light, these photothermal agents generate localized hyperthermia, which can effectively induce bacterial cell death by disrupting cell membranes, denaturing proteins, and impairing other critical cellular structures and functions. The thermal effect, typically reaching temperatures sufficient to cause irreversible damage to bacterial cells, offers a non-antibiotic strategy that is less likely to engender resistance—a significant advantage in the ongoing battle against multidrug-resistant organisms [6, 7]. For instance, the heat generated by photothermal agents can increase the permeability of bacterial membranes, thereby facilitating enhanced uptake of antibiotics. This combined effect not only improves the antimicrobial activity but also potentially reduces the required dosage of antibiotics, thereby mitigating the risk of resistance development and minimizing adverse effects associated with high-dose antibiotic therapy. Moreover, photothermal agents can be functionalized with targeting ligands that direct their action specifically to bacterial cells, thereby sparing healthy tissues from collateral thermal damage—a critical consideration in the clinical translation of this technology [8, 9].

The integration of diagnostic and therapeutic functions into a single nanoparticle platform, represents a cutting-edge advancement in nanomedicine. Caged gold nanostars are a prime example of this innovation, offering potential applications in targeted drug delivery and real-time imaging. The enhanced plasmonic properties of these nanostars facilitate better imaging contrast and more efficient photothermal therapy, making them a versatile tool in cancer treatment and other medical applications. Advanced characterization techniques are crucial for understanding the properties and behavior of nanoparticles. Techniques such as FT-IR, MALDI-TOF, and sp-ICPMS provide detailed insights into the composition, structure, and stability of nanoparticles. Stability tests, including those in different media and at varying temperatures, are essential to ensure that nanoparticles maintain their functional properties over time. The research on barley-synthesized nanoparticles demonstrates exceptional stability, which is critical for their practical applications. The advancements in nanoparticle research have significant implications for the field of drug delivery. Green-synthesized nanoparticles, with their biocompatibility and stability, are ideal candidates for drug carriers. Their ability to target specific cells and release drugs in a controlled manner enhances the efficacy and reduces the side effects of treatments. The antibacterial properties of AgNPs also offer potential for developing new antimicrobial therapies, particularly for infections that are resistant to conventional antibiotics. Caged gold nanostars, with their theranostic capabilities, represent a promising avenue for personalized medicine. These nanostars can be engineered to deliver drugs directly to cancer cells while simultaneously providing imaging feedback. This dual functionality not only improves the precision of treatments but also allows for real-time monitoring of therapeutic efficacy, paving the way for more effective and tailored medical interventions. Future research in this domain is likely to focus on optimizing the synthesis methods to enhance the yield and functionality of nanoparticles. Exploring other plant sources and understanding the underlying mechanisms of nanoparticle formation will contribute to the development of more efficient and sustainable synthesis techniques. Additionally, further studies on the interaction of nanoparticles with biological systems will be crucial for advancing their applications in biomedicine.

The integration of nanoparticles into existing drug delivery systems and the development of new photothermal platforms will continue to be a major area of interest. Innovations in this field have the potential to revolutionize medical treatments, making them more targeted, efficient, and less invasive. The cross-disciplinary collaboration between chemists, biologists, and medical professionals will be essential to fully realize the potential of nanoparticle-based therapies. The current state of research on green-synthesized nanoparticles and theranostic nanoplatforms highlights significant advancements and emerging trends. These innovations not only address critical issues such as antibiotic resistance and cancer treatment but also pave the way for sustainable and effective medical solutions. The ongoing exploration and development in this field promises to transform the landscape of nanomedicine and drug delivery systems.

Both studies, [1, 2] showcase innovative approaches to nanoparticle synthesis and application with distinct aspects of therapeutic applications demonstrated by each research gorup. The barley-mediated synthesis of gold and silver nanoparticles (AgNPs) leverages green chemistry principles, utilizing barley grains as a biological resource for producing biocompatible and stable nanoparticles. This method is environmentally friendly and cost-effective, reducing reliance on toxic chemicals typically used in nanoparticle synthesis. The resulting nanoparticles exhibit significant antibacterial activity, demonstrating their potential in medical and environmental applications. The study on caged gold nanostars (C-GNS) introduces a novel nanoplatform combining the plasmonic properties of gold nanostars with the structural advantages of hollow-shell architectures. This hybrid structure enhances the optical and photothermal properties of the nanoparticles, making them highly suitable for photothermal therapeutic applications. The synthesis methods developed are robust, providing high reproducibility and control over nanoparticle morphology and size distribution.

Despite these strengths, both studies exhibit certain limitations. The barley-mediated synthesis of nanoparticles, while eco-friendly, may face scalability issues. The precise control over nanoparticle size and uniformity remains a challenge, which could impact their efficacy and consistency in practical applications. Additionally, the biological components from barley can introduce variability in the nanoparticle synthesis process, affecting reproducibility across different batches. In the case of C-GNS, while the novel synthesis method and resulting properties are impressive, there are concerns regarding the complexity and cost of production. The integration of gold nanostars within a hollow-shell structure involves multiple synthesis steps, which could limit large-scale production and widespread adoption. Furthermore, the biocompatibility and long-term stability of C-GNS in biological systems require extensive evaluation to ensure their safety and effectiveness for in vivo applications.

The literature reveals several gaps that need to be addressed to advance the application of these nanoparticles [5, 10, 11]. For the barley-mediated synthesis, detailed studies on the interaction mechanisms between the nanoparticles and bacterial cells are necessary to fully understand their antibacterial action and potential resistance mechanisms. Moreover, investigations into the environmental impact and degradation of these nanoparticles are crucial for assessing their long-term sustainability and safety. Regarding C-GNS, further research is required to optimize the loading efficiency of therapeutic agents and to evaluate the pharmacokinetics and biodistribution of these nanoparticles in vivo [2]. There is also a need to explore the potential immunogenicity and toxicity of C-GNS to ensure they do not elicit adverse immune responses when used in clinical settings. The studies present some inconsistencies that warrant further investigation. For instance, the reported size and morphological characteristics of nanoparticles synthesized via barley grains exhibit variability depending on the characterization techniques used. This discrepancy highlights the need for standardized protocols and comparative studies to validate the findings [1].

In the context of C-GNS, there are debates regarding the optimal conditions for achieving the desired plasmonic and photothermal properties. The influence of various synthesis parameters on the structural integrity and functional performance of C-GNS needs to be systematically studied to resolve these controversies [2, 12, 13]. The implications of these findings are far-reaching for the fields of nanomedicine and environmental science. The barley-mediated synthesis approach exemplifies a sustainable method for producing functional nanoparticles, potentially revolutionizing the production of antimicrobial agents and environmental remediation tools [1]. The successful implementation of C-GNS in therapeutic applications could lead to significant advancements in cancer treatment, enabling precise imaging and targeted therapy with minimal side effects [2]. However, to translate these promising laboratory findings into real-world applications, addressing the identified gaps, inconsistencies, and potential risks is essential. Future research should focus on optimizing synthesis protocols, conducting comprehensive in vivo studies, and developing standardized testing frameworks to ensure the safety, efficacy, and scalability of these innovative nanoparticles. By doing so, the scientific community can harness the full potential of these technologies to achieve impactful advancements in healthcare and environmental sustainability. The research conducted in both studies provides a comprehensive understanding of the synthesis and applications of gold and silver nanoparticles, but it also highlights a noticeable gap and an area of potential overlap that could inform future investigations. By comparing and contrasting the findings of these two studies, it is possible to identify opportunities for practical applications and areas for further research, especially in the context of drug delivery systems.

In the context of AgNPs synthesized via barley-mediated green chemistry, the issues related to the ease of aggregation and dissociation in solution are of critical importance and warrant detailed discussion. Barley extract, serving as both a reducing and capping agent, plays a pivotal role in determining the stability of the synthesized nanoparticles. The tendency of AgNPs to aggregate is a significant challenge, primarily due to the high surface energy of nanoparticles, which drives them to minimize their surface area by coming together, leading to agglomeration. This process not only diminishes the colloidal stability of the nanoparticles but also adversely affects their biological activity and functionality, particularly in therapeutic applications where particle size is a crucial determinant of cellular uptake and biodistribution. Barley extract, rich in polyphenolic compounds, proteins, and polysaccharides, acts as a stabilizing agent by forming a capping layer around the AgNPs, thereby preventing their aggregation. The phenolic groups in barley extract provide steric hindrance and electrostatic stabilization, which help maintain the dispersion of nanoparticles in solution. However, the effectiveness of barley extract in preventing aggregation can vary depending on the concentration of the extract, the pH of the solution, and the ionic strength of the medium. These factors influence the thickness and density of the capping layer, which in turn affects the interparticle forces. For instance, at lower concentrations of barley extract, the capping layer may be insufficiently dense, leading to inadequate stabilization and a higher propensity for aggregation. Conversely, at higher concentrations, excessive capping can lead to oversaturation, where the nanoparticles might still aggregate due to Van der Waals forces overcoming the repulsive forces provided by the capping agents. Furthermore, the dissociation of AgNPs, or the breakdown of aggregated nanoparticles back into their individual forms, is another aspect that needs consideration. Barley extract's ability to facilitate the redispersion of agglomerated nanoparticles could be critical in maintaining the functional integrity of the nanoparticles over time. This redispersion is often influenced by the reversibility of the capping layer's interaction with the nanoparticle surface, which could be modulated by changes in environmental conditions such as pH, temperature, and the presence of competing ions or molecules in the solution. The dynamic nature of the barley extract as a capping agent thus presents both an advantage and a challenge—while it can stabilize nanoparticles and prevent aggregation, it must also be sufficiently robust to prevent dissociation under physiological conditions [1, 14].

So, technically, it can be stated that the barley-mediated synthesis of AgNPs introduces a complex interplay between aggregation and dissociation dynamics, which are critically influenced by the nature of the capping provided by barley extract. Addressing these issues requires a nuanced understanding of the chemical interactions at the nanoparticle interface, and the ability to fine-tune these interactions to optimize the stability and functionality of the AgNPs for specific applications.

The primary gap between the two studies lies in their focus and application. The assigned group by this commentary, [1], emphasizes the antibacterial potential of AgNPs synthesized using barley grains. This research is grounded in the green synthesis approach and highlights the sustainable production of nanoparticles with significant antimicrobial properties. However, the study does not delve deeply into the potential applications of these nanoparticles in drug delivery systems, which represents a crucial gap considering the extensive interest in using nanoparticles for targeted therapeutic delivery. While the study of C-GNS, [2], explores the theranostic applications of caged gold nanostars (C-GNS) with a particular emphasis on their potential for photothermal therapy and sensing applications in oncology. Both can overlap in the field of biological activity which encourages us to think about the integration of the strategies of drug delivery design engineering done by each group. While this study provides a detailed exploration of the multifunctionality of gold nanostars in medical applications, it does not investigate the antibacterial properties of these nanoparticles, nor does it explore the green synthesis approach. The overlapping area between the two studies is the inherent potential of both types of nanoparticles (gold and silver) to be used in biomedical applications. Both studies recognize the importance of nanoparticle stability, biocompatibility, and their functional capabilities in medical settings. This overlap suggests a combined approach where the green synthesis methods used in [1] could be adapted to produce AuNPs similar to those in [2], potentially leading to eco-friendly and multifunctional nanoparticles that could address both antibacterial and theranostic needs.

The practical applications of combining the green synthesis of nanoparticles with their use in drug delivery systems are vast. For instance, the barley-derived AgNPs could be engineered for targeted drug delivery in antibacterial treatments. Their proven efficacy against pathogens like Pseudomonas aeruginosa and Escherichia coli at low concentrations underscores their potential for treating bacterial infections without causing significant cytotoxicity to human cells. Additionally, the inherent biocompatibility of these nanoparticles, due to their green synthesis, further supports their use in clinical settings. On the other hand, the gold nanostars could be optimized for drug delivery in cancer treatment. Their unique plasmonic properties and the ability to induce localized hyperthermia make them ideal candidates for photothermal therapy. By incorporating green synthesis methods, the production of these nanostars could become more sustainable and potentially more cost-effective, making advanced cancer treatments more accessible.

In terms of the functionality, the dual effect of plant extracts in nanoparticle synthesis—particularly their inherent bacteriostatic properties and inhibitory effects on cancer cells—presents a promising avenue for enhancing the therapeutic potential of caged gold nanoparticles (C-GNS). Plant extracts are known for their rich composition of bioactive molecules such as polyphenols, flavonoids, and alkaloids, which confer potent antimicrobial and anticancer activities. When these extracts are used in the synthesis of C-GNS, the resulting hybrid nanostructures not only benefit from the biocompatibility and plasmonic properties of gold but also inherit the therapeutic properties of the plant extracts. The bacteriostatic properties of plant extracts, attributed to their ability to disrupt microbial cell walls, interfere with protein synthesis, and inhibit nucleic acid function, can be synergistically enhanced when combined with C-GNS. The gold component of C-GNS, known for its photothermal properties, can be activated by near-infrared (NIR) light to generate localized heat, further amplifying the bacteriostatic effect through hyperthermia-induced bacterial cell death. This dual-action mechanism offers a multifaceted approach to combating bacterial infections, particularly in drug-resistant strains where traditional antibiotics may fail. The incorporation of plant-derived phytochemicals in C-GNS can thus provide an additional layer of antimicrobial defense, potentially reducing the required dosage of AuNPs and minimizing associated cytotoxicity [1, 2, 15–17].

On the other hand, AuNPs and AgNPs exhibit antibacterial activity primarily through several mechanisms. For example, the ability of AuNPs to induce oxidative stress within bacterial cells is well-documented. When AuNPs come into contact with bacterial cells, they catalyze the production of reactive oxygen species (ROS) such as superoxide anions, hydroxyl radicals, and hydrogen peroxide. These ROS can cause significant damage to cellular components, including lipids, proteins, and DNA, leading to cell death. The high surface-to-volume ratio of AuNPs enhances their interaction with bacterial cells, increasing the likelihood of ROS generation. Another key mechanism involves the direct interaction of AuNPs with the bacterial cell membrane. The physical interaction between AuNPs and the bacterial cell wall can lead to membrane disruption. This disruption results in the leakage of cellular contents and loss of membrane integrity, ultimately leading to bacterial cell death. The size and shape of AuNPs influence this interaction, with smaller nanoparticles and those with particular shapes (e.g., nanorods) exhibiting enhanced antibacterial properties. AuNPs can also interfere with intracellular processes by binding to essential biomolecules. This interaction can inhibit critical cellular functions such as DNA replication and protein synthesis. For instance, AuNPs have been shown to bind with bacterial DNA, disrupting its replication and transcription processes. Additionally, the surface functionalization of AuNPs can be tailored to enhance their binding affinity for specific bacterial targets, further increasing their antibacterial efficacy [18, 19].

In parallel, upon suggested integration between the two technical works of each group, the anticancer potential of plant extracts, which has been extensively documented in the literature, can be effectively harnessed in C-GNS formulations. The bioactive compounds in plant extracts exert their anticancer effects through various mechanisms, including the induction of apoptosis, inhibition of angiogenesis, and suppression of metastasis. When these compounds are conjugated with C-GNS, the resulting nanocomplexes can target cancer cells more effectively due to the enhanced permeability and retention (EPR) effect. Furthermore, the ability of C-GNS to deliver these bioactive molecules directly to the tumor site, combined with the photothermal effect of gold under NIR irradiation, can result in a powerful theranostic, and photothermal-based therapeutic nanoplatform that simultaneously diagnoses and treats cancer. This approach not only improves the selectivity and efficacy of cancer treatment but also reduces the side effects typically associated with chemotherapy and radiation therapy.

In conclusion, the integration of plant extracts' bacteriostatic and anticancer properties with the advanced capabilities of C-GNS represents a significant step forward in the development of multifunctional nanotherapeutics. This strategy not only leverages the natural therapeutic potential of plant extracts but also enhances the performance of AuNPs in biomedical applications. Future research should focus on optimizing the synthesis protocols to maximize the therapeutic synergy between plant extracts and C-GNS, and on conducting comprehensive in vivo studies to validate the efficacy and safety of these hybrid nanoparticles in clinical settings. Additionally, exploring the integration of green synthesis methods with advanced nanostructure designs could lead to the development of new, sustainable nanomaterials with enhanced performance and broader applicability. In summary, the studies reviewed provide a solid foundation for future innovations in nanoparticle synthesis and application. By bridging the gap between sustainable production methods and advanced nanostructure designs, researchers can develop next-generation nanomaterials that are both environmentally friendly and highly functional, paving the way for new advancements in medicine, environmental science, and beyond.

## Data Availability

No datasets were generated or analysed during the current study.
